# Supplementary material for: Uninterrupted optical resolution of identical point scatterers undergoing nanometric changes in distance
Source: Proc Natl Acad Sci U S A. 2026 Jul 13;123(29):e2604558123. doi: 10.1073/pnas.2604558123 (PMC13389594; doi:10.1073/pnas.2604558123)
Supplement: Supplementary file 1 — Appendix 01 (PDF) [file pnas.2604558123.sapp.pdf]

Supporting Information for

Uninterrupted optical super-resolution of identical point scatterers undergoing nanometric changes in distance

Thomas A. Hensel[1], Ole L. Schwarz[3], Tim Karrasch[2], Kerstin Göpfrich[2], and Stefan W. Hell[1,3*]

[1]*Department of NanoBiophotonics, Max Planck Institute for Multidisciplinary Sciences, Am Faßberg 11, 37077 Göttingen, Germany*
[2]*Heidelberg University, Center for Molecular Biology of Heidelberg University (ZMBH), Berliner Straße 45, 69120 Heidelberg, Germany*
[3]*Department of Optical Nanoscopy, Max Planck Institute for Medical Research, Jahnstraße 29, 69120 Heidelberg, Germany*

*Correspondence: Stefan W. Hell
Email: stefan.hell@mpinat.mpg.de

This PDF file includes:

Supporting text
Figures S1 to S13
Tables S1 to S2
Legends for Movie S1
SI References

Other supporting materials for this manuscript include the following:

Movie S1

Supporting Information Text

Materials and Methods

MINFLUX measurements

The MINFLUX tracking routine requires an initial COM estimate $x_{\mathrm{COM}}[0]$ of the fluorophores that have been attached to the DNA origami. This initial estimate is obtained by fitting a 2D-Gaussian to the area around the locally brightest spots in a Gaussian kernel smoothed confocal scan. The field of view of the confocal scans was 10 x 10 µm², with a pixel size of 50 x 50 nm², acquired with a dwell time per pixel of 100 µs and a 1-5 µW laser intensity.

The MINFLUX tracking routine is implemented by probing three fluorescence photon counts $[n_-, n_0, n_+]$ at positions $[-L/2, 0, +L/2]$ relative to $x_{\mathrm{COM}}[t]$ along the x and y direction. The new COM estimate is then calculated independently for each direction according to

$$x_{\mathrm{COM}}[t + \mathrm{d}t] = x_{\mathrm{COM}}[t] + \frac{L}{4} \frac{n_- - n_+}{n_+ + n_- - 2n_0},$$

which is derived from a parabolic fit to the three intensity values in a single direction. The position is not updated if fewer than five photons are collected, or if $2(n_+ + n_- - 2n_0) < |n_- - n_+|$, which limits the allowed absolute COM corrections to $L/2$ and excludes fits with negative curvature.

To account for the greater uncertainty of the initial COM estimates, a zoom-in approach was chosen. This started with an initial L of 200 nm, which was then reduced to 30 nm in a pre-programmed manner over the first ten localizations (3x L=200nm, 4x L=100nm, 3x L=60nm). The remaining localizations were acquired with an L of 30 nm. The time per xy localization $\mathrm{d}t$ was set to 0.63 ms or 0.98 ms to capture the typical transition times of the DNA origamis arm. The laser intensity was adjusted so that the sum of all photons emitted by a single fluorophore per localization was between 15 and 50.

Sample preparation

Design of DNA origami structures

The DNA origami structure was based on earlier designs by Kopperger et al. (1) and Dreher et al. (2) and was adapted using cadnano (version 2.4) (3). The structure uses the p8064 scaffold and was folded into the desired square-shaped two-layer sheet using 188 oligonucleotide staple strands. Staples at the outer edges were omitted to reduce stacking interactions between separate DNA origami structures (2). A complete list of staple sequences is provided in Table S1, and the cadnano design is shown in Figure S 1.

On the top side of the two-layer sheet, two staples were extended to create binding sites for transient hybridization (binding site1/2; two thymidine spacers followed by the 7-nt sequence 5′-AGTCCTA-3′). These two sites were positioned to enclose a 90° angle with respect to a third extended staple carrying four thymidine spacers and a covalently linked Atto647N fluorophore (static fluorophore). A fourth staple, located at approximately equal distance from the two transient binding sites, was extended with three thymidine spacers followed by a 15-nt sequence forming the base of a flexible hinge (hinge base). Two additional strands completed the hinge: one carrying an internal Atto647N fluorophore (mobile fluorophore) and one containing a single-stranded sequence at the hinge terminus that is reverse-complementary to the transient binding-site sequence (hinge binding site). All modified strand sequences are listed in Table S2.

DNA origami assembly

DNA origami structures were assembled using 10 nM p8064 scaffold strand (tilibit nanosystems, Munich, Germany) in 10 mM Tris and 1 mM EDTA (TE buffer). Unmodified staple and hinge strands (Integrated DNA Technologies, Coralville, IA, USA; standard

desalting) and fluorophore-labeled strands (biomers.net GmbH, Ulm, Germany; HPLC purified) were dissolved in TE buffer and added at a fivefold molar excess relative to the scaffold. The folding mixture contained 1× Tris–acetate–EDTA (TAE; Sigma-Aldrich) and 20 mM $MgCl_2$ (Sigma-Aldrich). The origami structures were folded by thermal annealing in a thermocycler (Bio-Rad C1000 Touch), cooling from 70 °C to 20 °C over 12 h, followed by a 3 h hold at 40 °C and subsequent cooling to RT.

DNA origami purification

Folded structures were purified from excess staples by spin filtration using an Amicon Ultra centrifugal filter (100 kDa MWCO; Merck Millipore, UFC5100BK) in a Fresco 17 microcentrifuge (Thermo Scientific, 75002420) at 4°C. Filters were pre-rinsed by centrifugation with 500 µL of 1× TAE and 5 mM $MgCl_2$ for 5 min at 13,000 × g. Samples were loaded in two rounds by combining 25 µL DNA origami with 475 µL of 1× TAE and 5 mM $MgCl_2$ and centrifuging as above. DNA origami was recovered by inverting the filter into a fresh microtube and centrifuging for 2 min at 1,000 × g. The $MgCl_2$ concentration was then adjusted to 20 mM. Samples were stored for up to 5 days at 4 °C until imaging.

AFM imaging and analysis

To confirm successful folding, purified DNA origami samples were imaged by high-speed atomic force microscopy (AFM). Two microliters of purified sample were diluted in 48 µL imaging buffer (1× TAE, 20 mM $MgCl_2$), pipetted onto freshly cleaved mica (Sigma-Aldrich, AFM-71856-02), and incubated for 1 min. Subsequently, 1 mL imaging buffer was added to the imaging chamber. Imaging was performed on a JPK Nanowizard Ultra Speed AFM (Bruker) in AC mode using a FastScan-D cantilever (resonance frequency in water: 110 kHz; spring constant: 0.25 N/m; Bruker).

Images were processed using Gwyddion (version 2.63, (4)) by applying standard operations such as flattening, removal of faulty lines and artifacts, and the manual adjustment of color scales (Figure S 2).

Cleaning process of the cover slips

The coverslips (170 µm, No. 1.5H, 18 x 18 mm², Paul Marienfeld GmbH & Co. KG) were cleaned by incubating them in acetone, followed by isopropanol and then double-distilled water, for 5 minutes each. After removing them from the double-distilled water bath the coverslips were dried using a nitrogen flow. No more than five days before a measurement, the coverslips were plasma cleaned using oxygen for 5 minutes at 200 W.

Construction of flow chamber

A flow channel was made to allow the exchange of reagents and buffers during sample preparation. The flow channel was prepared by attaching two strips of scotch tape (Scotch double-sided tape, 3M) to a microscopy slide at a distance of about 5 mm. The channel was then closed by placing a previously cleaned coverslip on top. Finally, any excess tape was removed using a scalpel.

Preparation of the DNA origami sample

To prepare a sample for imaging, the flow chamber was first washed with TAE buffer and the concentration of $MgCl_2$ chosen for the measurement. A solution of the DNA origami was then flushed in and incubated for 5 min. The dilution of the DNA origami solution was chosen so that the final surface density of origami was about 0.1 μm^{-2}. After the incubation period the unattached origami were flushed out of the channel by adding the imaging buffer. The imaging buffer was based on TAE buffer with the corresponding concentration MgCl2, 10% glucose (D(+)-glucose monohydrate, cat. no. 6780.1, Carl Roth GmbH + Co. KG), 0.1 mM methyl viologen (cat. no. 856177, Sigma-Aldrich Chemie GmbH), 0.1 mM Trolox (cat. no. 23881, Sigma-Aldrich Chemie GmbH) and adding 1 µL of pyranose oxidase enzyme system per 100 µL of buffer. The pyranose oxidase enzyme system was prepared by solving 10 mg of pyranose oxidase (cat. no. P4234, Sigma-Aldrich Chemie GmbH), 170 µL PBS, 80 µL

catalase (cat. no. 190311, MP Biomedicals). In the end the slide was sealed with either epoxy glue (UHU 2x Plus Sofortfest, UHU GmbH & Co. KG) or picodent twinsil speed 22 (Picodent Dental-Produktions- und Vertriebs GmbH).

Data analysis and localization

The analysis segments, filters and processes localization traces to obtain the temporal state assignments of the DNA structure, system-related quantities like PSF parameters (contrast and brightness of illumination) and finally emitter positions (co-localizations). In the following, we give a conceptual summary of our analysis routine.

In a first segmentation step, a series of position-count tuples is first standardized and partitioned into quasi-stationary blocks using a change-point detection algorithm (5). Each block represents a segment in which the emission statistics are approximately constant. The parameters of the change-point detection are empirically chosen such as to yield break points at the bleaching steps of a trace or significant changes in brightness.

Second, we filter the obtained segments and initiate an iterative labelling routine that infers the number of molecules that are active in the current segment. To that end, robust descriptors are derived for each segment: average photon counts, SBRs, and the visibility/contrast of the emission signal. These features are used to identify high-confidence examples of background, single- and multi-fluorophore segments. A deliberately conservative approach is taken: only clear exemplars are labelled initially, and these seed labels are then propagated across the dataset with a k-means-based propagation algorithm. Outliers and ambiguous cases are aggressively excluded to maintain reliability of the assignment.

We then proceed to the calibration stage to obtain system parameters, such as the initial visibility of the illumination minimum ν_0 or expected single-molecule brightness and background levels. These values act as priors for subsequent model fits. Only traces with sufficient contrast are used for calibration, ensuring that the priors are robust.

Within blocks containing active emitters, a motility analysis is performed to capture dynamical behavior of the origami. Groups of contiguous blocks are examined for signatures of motion of the COM, and when present, a HMM is fitted to the time series of positions. In fact, we fit 10 randomly initialized HMMs for each number of states (ranging between 1 and 5) in order to remain agnostic about the apparent dynamics of the system. For each trace, the best model is selected according to a Bayesian information criterion. This yields per-tuple assignments of latent motility states together with statistics of the binding events, allowing quantification of dynamic transitions.

Last, we co-localize the two molecules with a MLE. The trace is bootstrapped into overlapping chunks, either according to a number of photons per bin or a time interval for the chunks and a specified stride to move the bin. Each chunk is fitted to a parametric PSF model under a Poisson likelihood, stabilized by the calibration priors. Overlapping chunks both increase temporal resolution and provide bootstrap-like uncertainty estimates.

Note that we apply our sliding-window estimator only on parts of the trace of the same state. We thereby avoid mixing states within a single estimation window. Such mixing may yield ambiguous and biased results (see in-silico study, Figures S3-S13).

After having obtained time-resolved emitter positions, we resolve ambiguities arising from the ill-behaving likelihood. Since the measurement remains ambiguous with respect to permutations and mirroring of the obtained positions, we sort and mirror the positions as to minimize their overall pair-wise distance.

In summary, the workflow proceeds as follows:

1. compress and segment raw traces at bleaching steps,

2. label segments conservatively with number of active molecules,
3. detect dynamical states and dwell times,
4. locally calibrate PSF-parameters and background against trusted single-molecule references, i.e. calibrate per trace if possible
5. infer positions of two molecules simultaneously with MLE.

Derivation of COM localization uncertainty and brightness

In order to derive the center-of-mass localization uncertainty for two fluorescent molecules with finite separation d and COM x_0, we consider a quadratic intensity profile of the form $y_i = a\, x_i^2$ to probe the markers at three distinct positions $x_i = \pm\frac{L}{2}, 0$. The expected response of the two markers reads

$$y_i = a\left(x_i - \left(x_0 - \frac{d}{2}\right)\right)^2 + a\left(x_i - \left(x_0 + \frac{d}{2}\right)\right)^2 + b,$$

where a denotes the normalization (brightness) of the incident beam and b is a background parameter. The mean intensity y_i of the i-th exposure can be simplified to

$$y_i = a\left(2(x_i - x_0)^2 + \frac{d^2}{2}\right) + b,$$

and the derivative with respect to x_0 is given by $\partial_{x_0} y_i = 4a(x_0 - x_i)$. From that, we can calculate the Fisher Information J with respect to the COM x_0 for a Poisson variable, near $x_0 = 0$:

$$J_{x_0} = \sum_i \frac{\left(\partial_{x_0} y_i\right)^2}{y_i} = \frac{8a^2L^2}{b + a\frac{L^2 + d^2}{2}}.$$

Since the localization uncertainty (Cramer Rao Bound) of x_0 is given by $\sigma_{x_0} = 1/\sqrt{J}$, we can calculate the ratio of the localization uncertainties $\sigma_{x_0}(d \neq 0)/\sigma_{x_0}(d = 0)$ from the ratio of Fisher Informations

$$\frac{\sigma_{x_0}(d \neq 0)}{\sigma_{x_0}(d = 0)} = \sqrt{\frac{J_{x_0}(d = 0)}{J_{x_0}(d \neq 0)}}$$

This readily yields

$$\frac{J_{x_0}(d = 0)}{J_{x_0}(d \neq 0)} = 1 + \frac{d^2}{L^2}\frac{SBR}{SBR + \frac{2}{3}}.$$

Similarly, we calculate the ratio of the expected mean intensity among the three exposures for either $d = 0$ and finite separation, which yields:

$$\frac{I_{fluor}(d \neq 0)}{I_{fluor}(d = 0)} = 1 + \frac{3d^2}{2L^2}\frac{SBR}{SBR + 1}.$$

In case the two emitters have different brightness γ_1 and γ_2, we define the brightness imbalance $\beta = \frac{\gamma_1 - \gamma_2}{\gamma_1 + \gamma_2}$. The ratio of apparent brightnesses then becomes

$$\frac{I_{fluor}(d \neq 0)}{I_{fluor}(d = 0)} = 1 + \frac{3d^2}{2L^2} \frac{SBR}{SBR + 1} (1 - \beta^2).$$

Consider a brightness imbalance of 10%, this will only attenuate the apparent brightness by 1% due to the quadratic suppression of β.

We can further generalize this result to 2D by assuming an isotropic angular distribution of emitters separated by a fixed distance d. Since the apparent brightness scales quadratically in d, projecting d along each axis of the probing pattern results in an average reduction of the apparent brightness by a factor of two (since $\langle \cos^2 x \rangle = \langle \sin^2 x \rangle = 1/2$):

$$\frac{I_{fluor}(d \neq 0)}{I_{fluor}(d = 0)} = 1 + \frac{3d^2}{4L^2} \frac{SBR}{SBR + 1} (1 - \beta^2).$$

Simulation of spatio-temporal resolution

The precision of the co-tracking of two fluorophores and identification of states with respect to the estimated positions depends on a large number of parameters, such as the number of available photons, the SBR, the geometry of the probing pattern (orientation(s) of line-shaped minimum, number of exposures and scaling parameter L), the distance between the fluorophores themselves, the baseline-distance, magnitude and duration of a distance change. Further, parameters of the analysis such as the chosen window size for the estimator, may influence the estimation process. In the following, we present a simulation study of the most common parameters to exemplify their influence on the quality of this method.

Spatio-temporal resolution and bias

To study temporal resolution in a controlled setting, we implement a deterministic change in distance between two fluorophores. The baseline distance was 13 nm, the elevated distance was 22 nm, the total trace length was 150 ms, and the change occurred at 50 ms. We refer to these scenarios as "pulses". Pulse durations of 1, 5, 10, 15, 20, 30, and 40 ms were simulated with 50 replicates (traces) per duration, using a photon budget of 100 collected photons per illumination pattern, SBR = 10, a 10 ms estimation window, and a 1 ms stride. In essence, we observe that a finite window smooths the estimates (Figure S 3), appears unbiased for appropriately chosen window sizes (Figure S 4, Figure S 5) and introduces a temporal lag depending on the window size (Figure S 6).

To examine the effect of the magnitude in distance changes (pulse amplitude), we simulated 40 ms pulses from a 13 nm baseline with amplitudes of 1, 5, 10, 15, and 20 nm, corresponding to high-distance states of 14, 18, 23, 28, and 33 nm. These conditions were combined with photon budgets of 25-100 photons per pattern, using a 10 ms window, a 1 ms stride, and 50 replicates per condition (see Figure S 7). The results allow to infer the required number of photons in order to detect a pulse of certain amplitude reliably.

Asymptotically, the quality of the estimates depends on the number of collected photons with a regular shot-noise scaling. However, for low photon number, the estimates may not converge or be biased, as the MLE is only asymptotically unbiased. We investigate the dependence of the (relative) estimation error of the pulse magnitude as well as the (relative) classification error of the states (small distance, high distance) with respect to the available photon number per pattern. Hence, the same 13-to-22 nm pulse geometry was used, but the photon budget and pulse duration were varied jointly. Photon budgets of 25, 50, 75, and 100 photons per pattern were combined with pulse durations of 1-40 ms, using a 10 ms window and 1 ms stride, with 50 replicates per grid point (see Figure S 8).

To assess potential directional bias of the distance estimate with respect to the relative orientation of two-fluorophore vectorial distance and the orientation of the probing pattern, we repeated the walking simulations for 15 equally spaced in-plane directions between 1 and 180 degrees and for two PSF types, namely a line-shaped minimum (TEM-like mode) and a rotationally symmetric "doughnut" excitation, while keeping the photon budget, background level, trace duration, and estimator window fixed (see Figure S 9).

Simulation of the dynamic DNA origami

Based on the previous results, we simulate the diffusive origami construct. In particular, we show that the use of a soft prior for the position of the fixed fluorophore can be avoided when more orientations of the illumination pattern are available. The experimental results presented in this paper were obtained with a scanning pattern consisting of 6 exposures aligned in a cross-like shape in 2D, where the three x-exposures have a y-oriented minimum and vice versa. This leads to ambiguities in the estimation process, as the likelihood function exhibits some symmetries. Therefore, the likelihood of estimated positions is invariant to mirroring and permutations of molecule positions. For a two-molecule system with a single fixed orientation, this can be solved by permuting and mirroring positions such as to reduce a

global RMSE of localizations. For three orientations and distances however, this problem cannot be solved easily. We therefore chose to inject information about the fixed fluorophore from the single molecule traces, where available. To show this effect, we simulate a dynamic DNA origami with the same kinetics as observed in our experiments (unbound state dwell times of 3ms, bound state dwell times of 15 ms) and assume 100 photons per pattern, an SBR of 10. Choosing our “degenerate” probing pattern, we observe the clustering of localizations at mirrored and permutated positions (Figure S 13). If we rotate the two inner exposures by $\frac{\pi}{4}$, we can avoid this problem completely (Figure S 12).

We furthermore note that segmenting the traces on the raw COM localization data and estimating the localizations of the two fluorophores on state-specific subsets of the trace improves estimation quality. This is because a sliding-window estimator operating on the full trace will mix the localizations of multiple states and try to estimate the localizations on this average. In this case the results will be difficult to interpret (Figure S 13). If, instead, we segment the trace before estimation, we can concatenate segments of the same state and estimate the properties of the state with a sliding window on the state-specific parts of the trace. In a subsequent step, we re-shuffle the obtained segments to obtain the properly ordered time series of estimates for the full trace (see Figure S 12 and Figure S 13).

Application: In-silico study of kinesin stepping dynamics

As a practically relevant scenario we emulate the well-known walking of the dimeric motor protein kinesin on a microtubule. We assume two identical dyes, each attached to the two microtubule-binding motor domains (heads) of the kinesin dimer. To simulate a realistic MINFLUX tracking sequence, we follow the moving protein and record photon counts according to the temporary distance of the fluorophores under random stepping dynamics. In essence, we investigate the use of two continuously emitting point emitters attached to the two kinesin heads and generate synthetic MINFLUX measurements from their joint signal. Unless stated otherwise, each condition comprises 50 independent traces of 500 ms duration sampled on a 1 ms time grid. Stepping is modelled with a mean dwell time of 20 ms and a nominal step size of 16 nm. In the default walking simulations, the motion follows the well-investigated hand-over-hand scheme, so that the inter-emitter distance stays constant. Following the procedure described above, we can recover the stepping dynamics of kinesin with our single-color co-tracking approach (Figure S 10). Moreover, we are able to recover the structural dynamics of a fictuous system undergoing random changes in distance between 0 nm and 16 nm, imitating an opened/closed conformational change (Figure S 11).

Figures

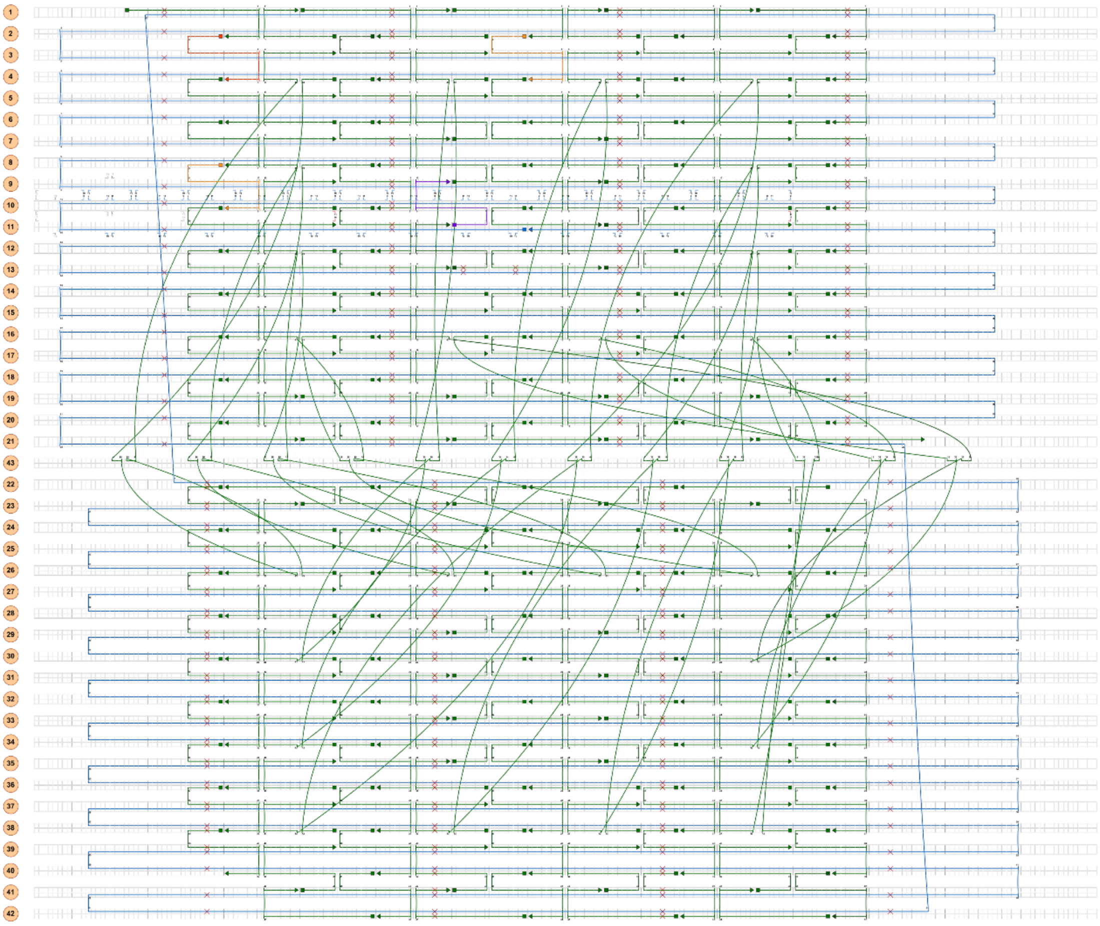

Figure S 1: ***Cadnano design of the DNA origami.*** *The p8064 scaffold strand is shown in blue. Orange staples are extended at the 5′ end by two thymidine spacers followed by seven unpaired nucleotides (5′-AGTCCTA-3′), forming transient binding sites. The red staple is extended at the 5′ end by four thymidine spacers followed by an Atto 647N dye. The purple staple is extended at the 3′ end by three thymidine spacers and 15 nucleotides that form the base of the hinge. The design was adapted from (1,2).*

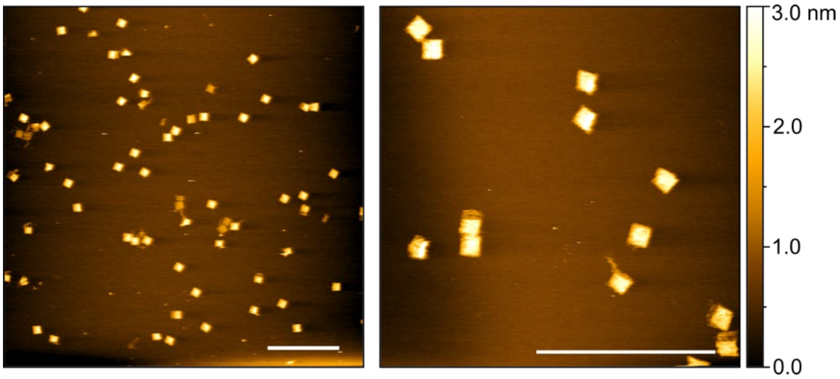

Figure S 2: ***Atomic force microscopy images of annealed and purified DNA origami structures.*** *Scale bars: 500 nm.*

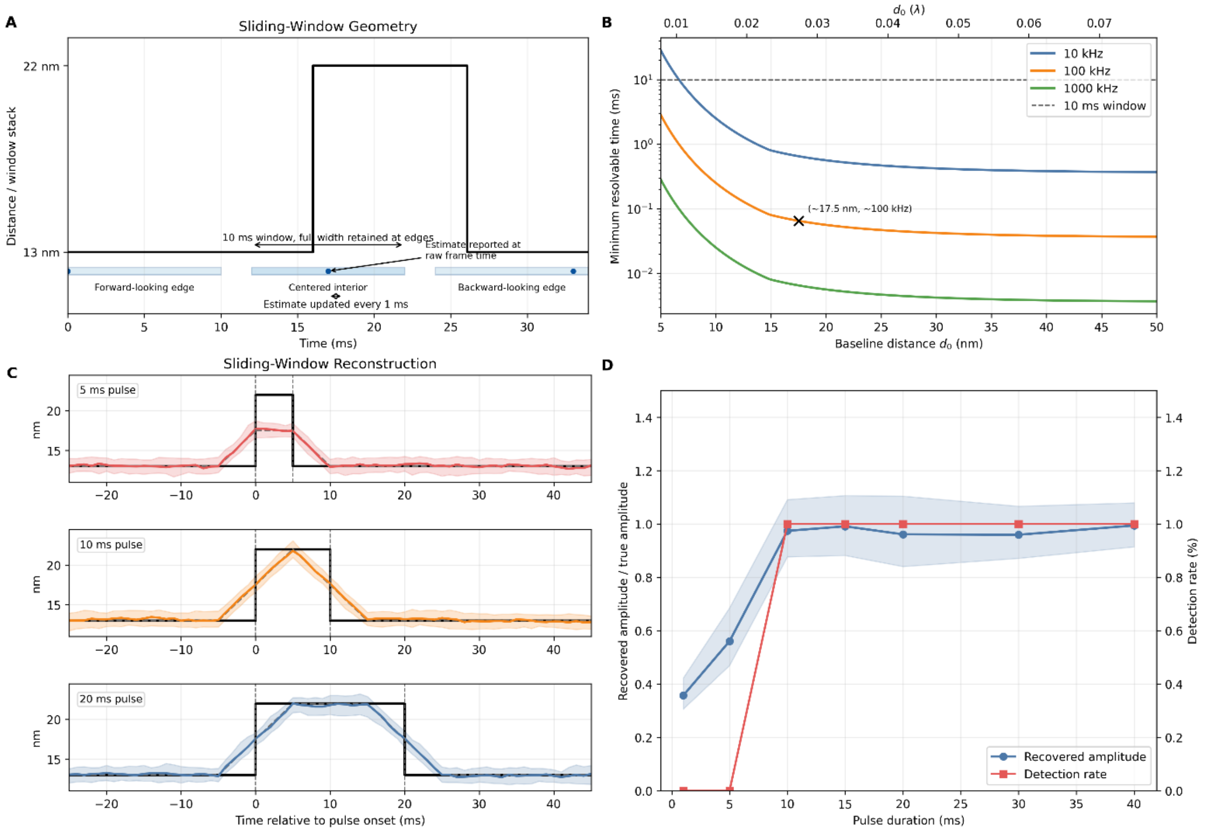

Figure S 3: Spatio-temporal resolution with respect to chosen window-size. A: a fixed window of 10 ms is chosen to resolve pulses of different duration. We adjust the anchor point of the window such that we can recover a trace of estimates with the same length as the original trace (forward, centered, backwards-looking window). B: theoretical prediction of the spatio-temporal resolution for the current scenario. C: the 10 ms second window smoothes short pulses, leading to a biases estimate of the distance towards the baseline distance (can be higher or lower). D: As soon as the pulse size reaches the window size, estimation can be performed reliably. (see also Figure S 7, Figure S 8)

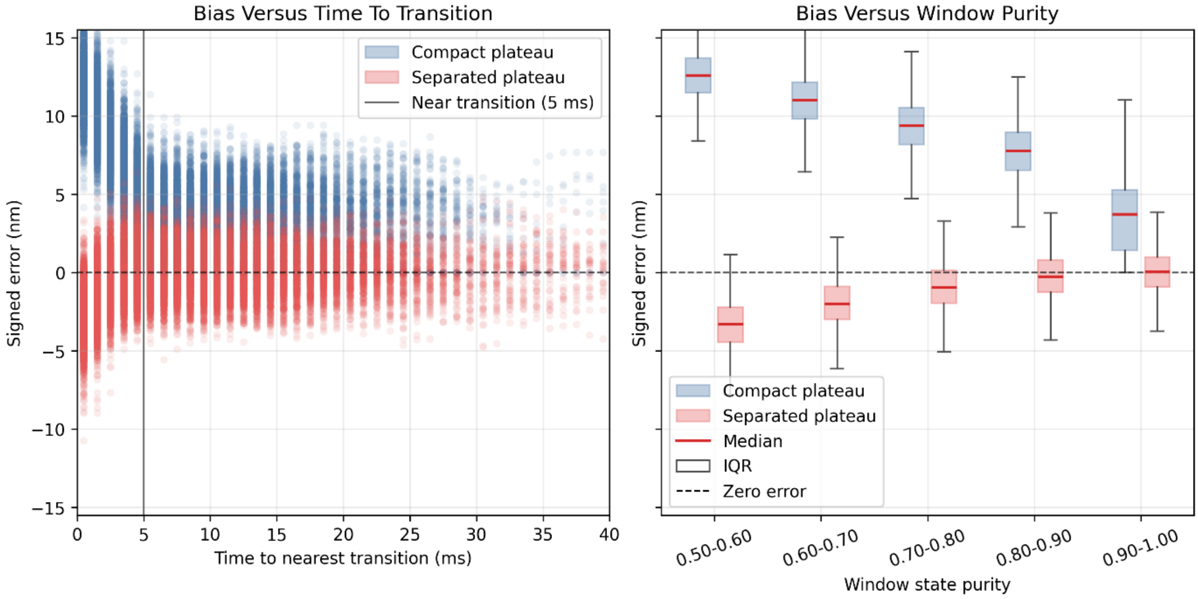

Figure S 4: Estimation bias for finite sizes of the estimation window. Left: Estimates are biased at the transition point. Right: For steady-state estimation the method is unbiased.

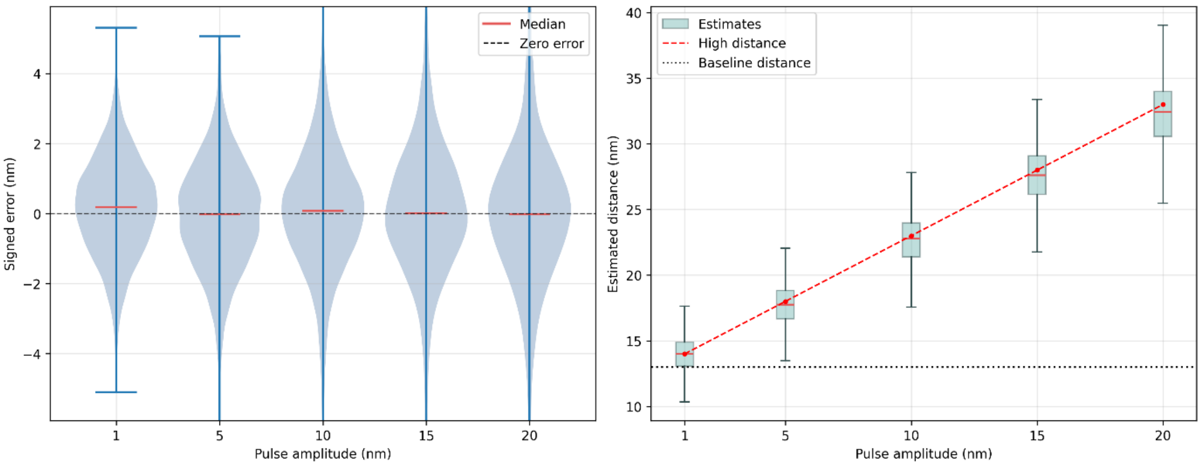

Figure S 5: Estimation bias with respect to a step-like amplitude change for a fixed estimation window. The estimation of various step-like changes in distance appears unbiased for the current method. (see Figure S 6 for step-like distance changes)

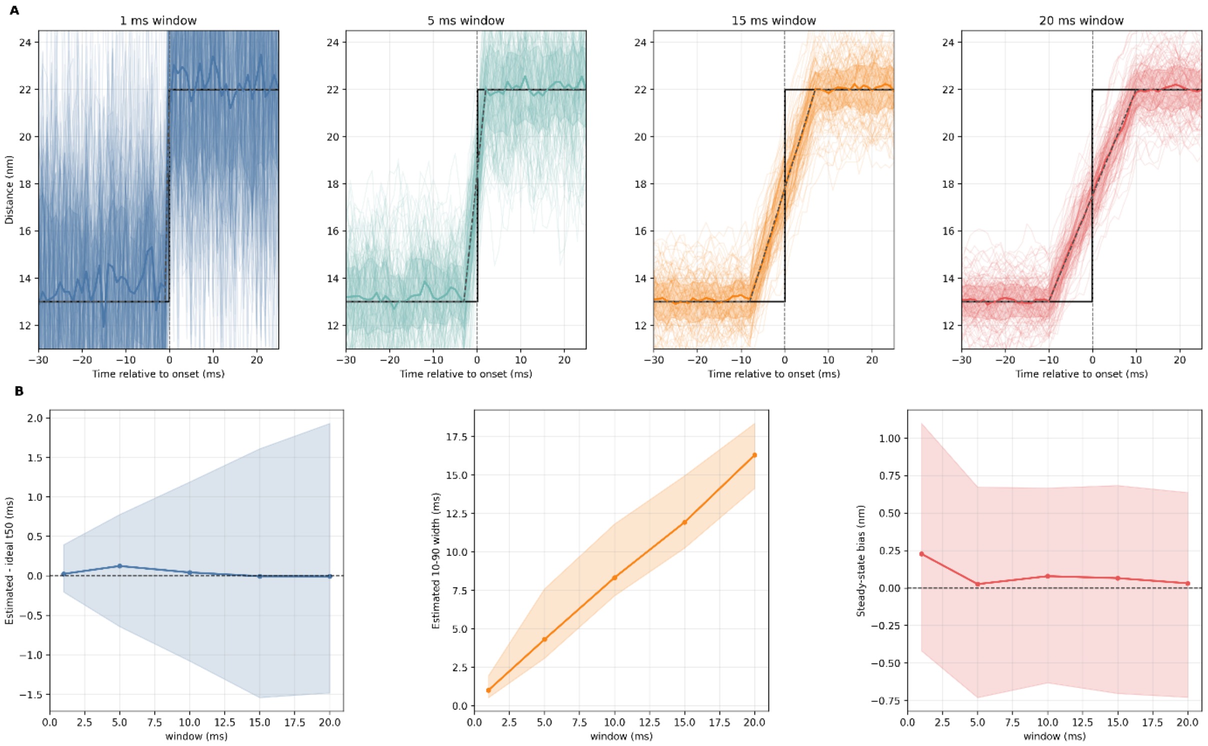

Figure S 6: Spatio-temporal resolution and temporal lag of estimates due to finite estimation windows. A: analyzing the same step-like distance change with different window-sizes yields the expected smoothing of the estimates. B: (left and center) larger estimation windows introduce an increasing lag in the rise-time of the detected distance change, as expected. (right) once the steady-state has been reached, the estimates appear to be unbiased.

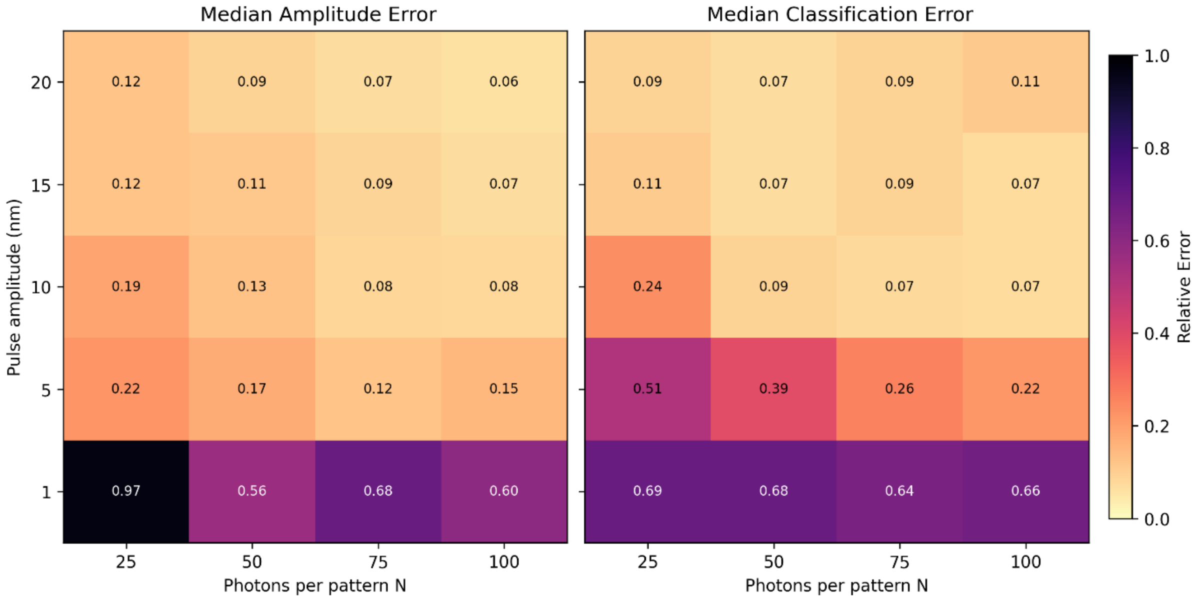

Figure S 7: Resolving pulse amplitudes. The resolvability of pulses depends on their amplitude and duration. Here we fix the pulse duration at 40 ms and only vary the magnitude of the pulses. We collect 25 to 100 photons per pattern at an SBR of 10. Pulses with an amplitude of 5-10 nm can be resolved reliably as apparent from the low error on the amplitude as well as the high classification accuracy of the state. Note that these results are valid for a 10 ms window and would change for other window sizes.

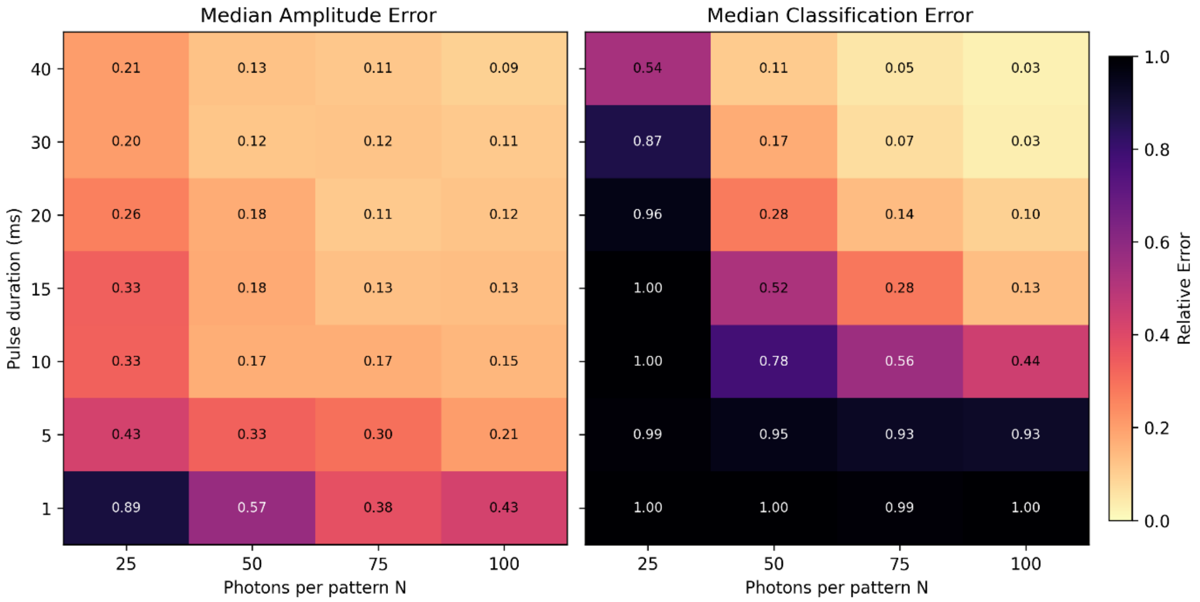

Figure S 8: Resolving pulse durations. The resolvability of pulses depends on their amplitude and duration. Here we fix the pulse amplitude at 9 nm and only vary the duration of the pulses. We collect 25 to 100 photons per pattern at an SBR of 10. Pulses with a duration above 15 ms can be resolved reliably as apparent from the low error on the amplitude as well as the high classification accuracy of the state. Note that these results are valid for a 10 ms window and would change for other window sizes.

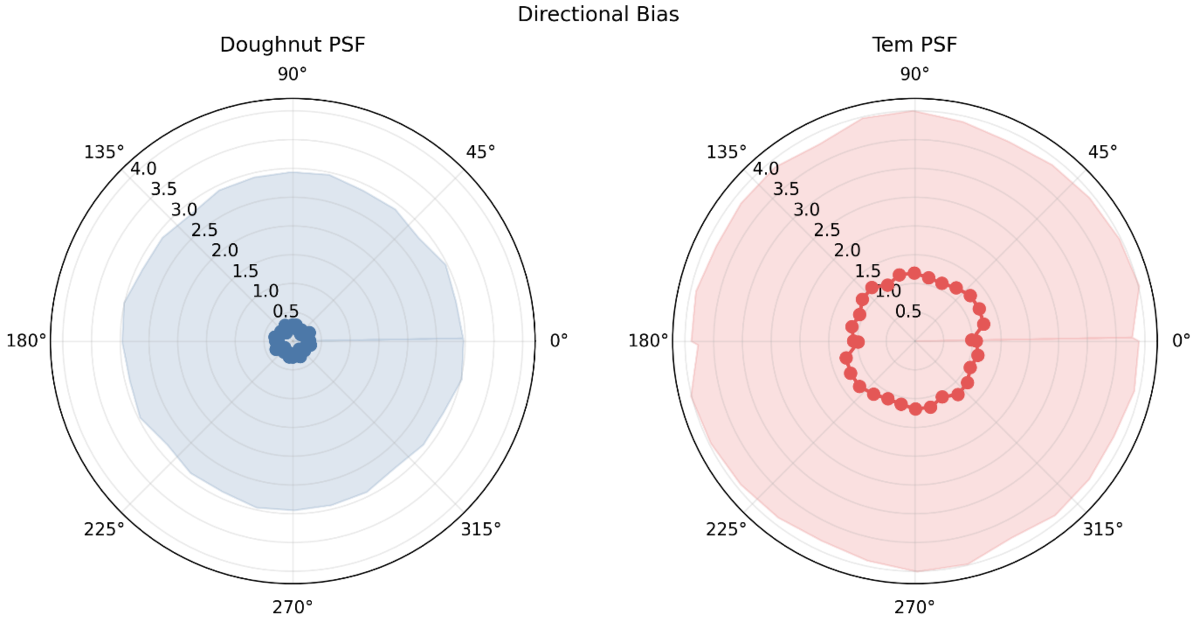

Figure S 9: Directional bias (RMSE) of vectorial distance estimation. Using a rotationally symmetric illumination field such as the "doughnut" mode yields a low estimation bias, but high angular uncertainty, since the directional information is lost due to the symmetry of the probing beam. The line-shaped minimum illumination field yields a slightly higher isotropic error for the distance, but allows precise angular resolution.

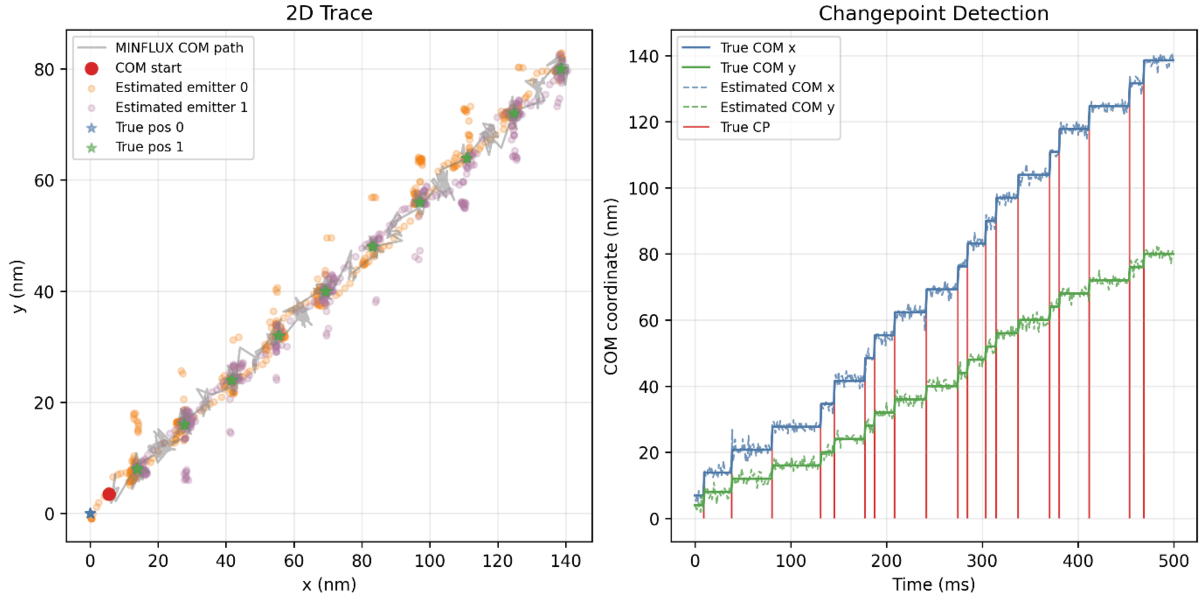

Figure S 10: Simulation of walking kinesin and segmentation of the trace. Left: Spatial trace of walking kinesin with reconstructed head (motor domain) positions from the current method. Artefacts due to the degenerate scanning patterns are visible as cross-hair-like localizations (compare S13). Right: COM coordinates and extracted steady-state segments. True steps are displayed as red lines, coinciding with the steps found by the current method.

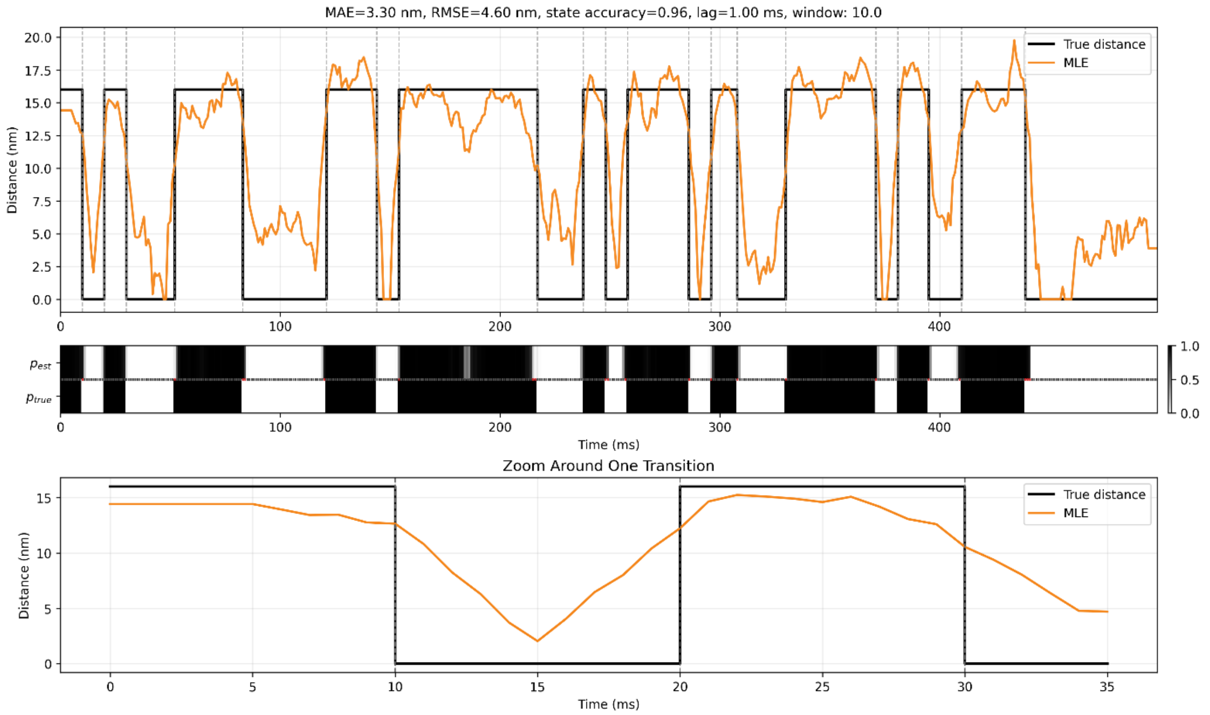

Figure S 11: Example of a macromolecule undergoing structural changes. The present method is able to recover distances of 0 and 16 nm consistently when switching between the two states.. Choosing a 10 ms window results in the smoothing of short transitions.

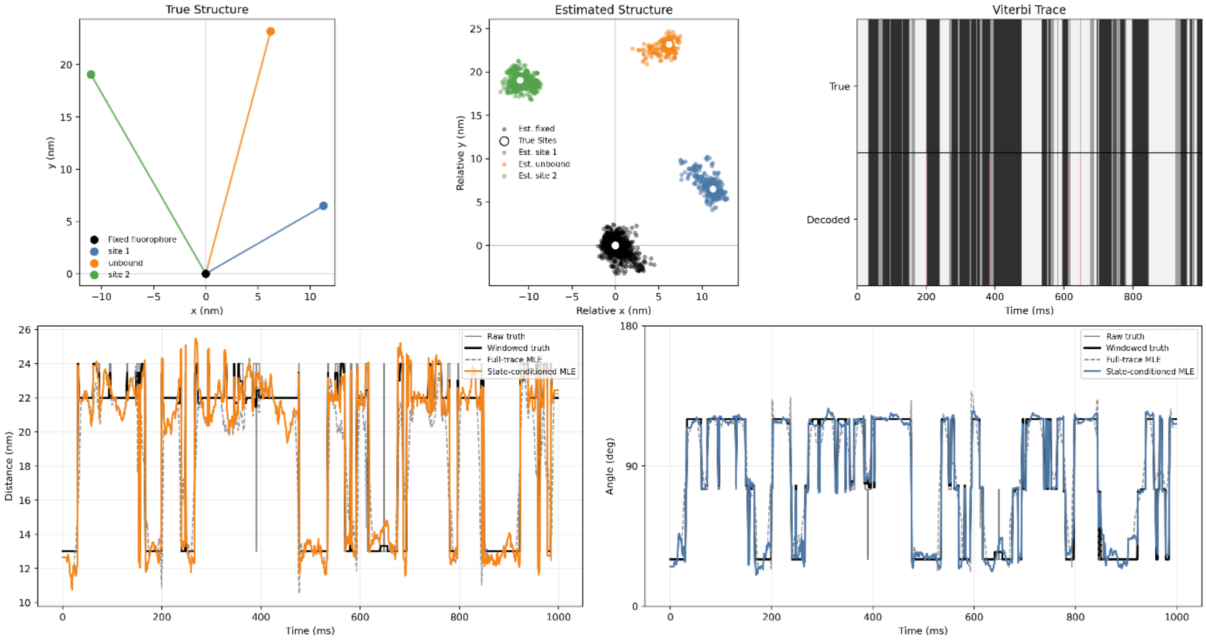

Figure S 12: Recovery of origami structure with improved scanning pattern. ***Top****: (left) ground truth structure as per the design of our experiment of the DNA origami. (center) estimated positions for the two fluorophores. No constraints were applied for the estimation. (right) states have been recovered correctly from the COM tracking data when compared to the ground truth. False assignments are highlighted with red color.* ***Bottom****: Estimated distances. Ground truth distance is depicted as grey line, the windowed-ground truth as solid black line. Estimating the distance on the already segmented trace (per state) allows to recover distances even for short-lived events (orange line). Applying a naïve sliding-window on the time-series of localizations mixes events of different states and compromises the estimation quality (grey dashed line). Similar observations are made for the angle estimate on the time-series (left). N=100 photons per pattern, SBR=10, mean dwell times: 15 ms (bound states), 3 ms (unbound state).*

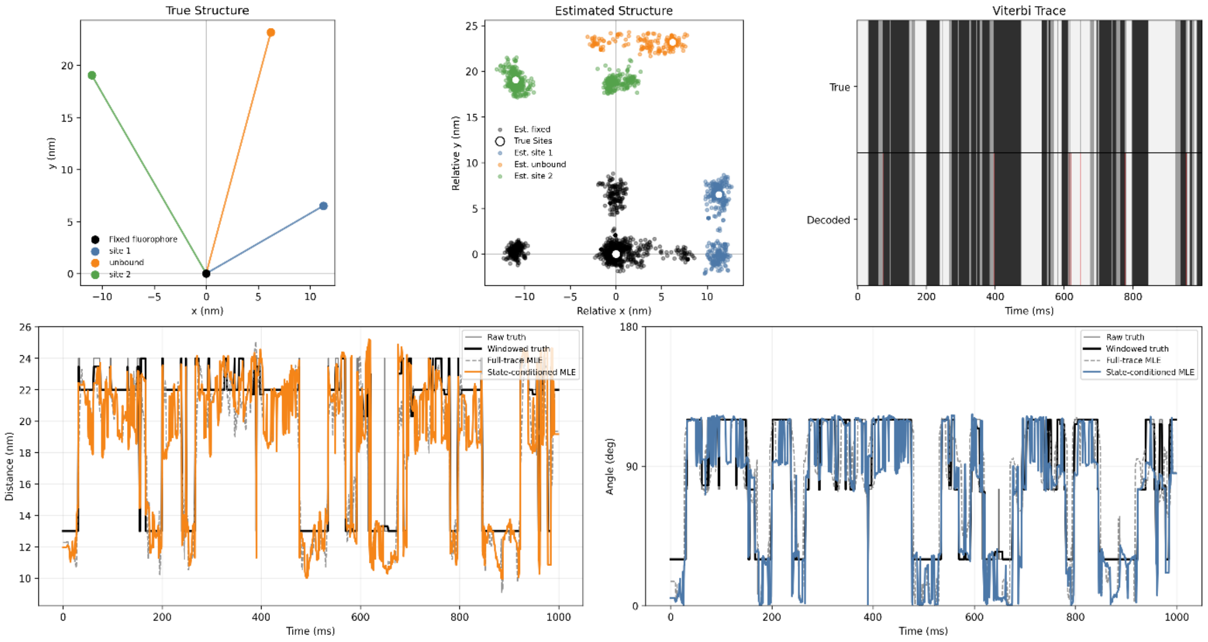

Figure S 13: Recovery of origami structure with a degenerate scanning pattern. ***Top***: *(left) ground truth structure as per the design of our experiment of the DNA origami. (center) Estimated positions for the two fluorophores. No constraints were applied for the estimation. Localizations include mirrored and permutated variants, making a reconstruction of the structure more difficult. (right) states have been recovered correctly from the COM tracking data when compared to the ground truth. False assignments are highlighted with red color.* ***Bottom***: *Estimated distances and angles. Compared to Figure S 12, the quality of the estimates is more unstable and exhibits a larger variance. N=100 collected photons per pattern, SBR=10, mean dwell times: 15 ms (bound states), 3 ms (unbound state).*

Tables

Table S1: Staple strands used to assemble the DNA origami sheet. Staple strand positions are given as B_H5′[p5′]_H3′[p3′], where the first helix number and bracketed index specify the staple's 5′ start (helix number and position within that helix), and the second pair specifies the staple's 3′ end. Sequences are listed in 5' to 3' direction.

Position	Sequence
B_41[56]_39[63]	TATCATTCAAATAAACAGCCATATAGAATAACATAAAAAC
B_21[152]_19[151]	GAGAATCGCTGAGAGACTACCTTTCGCTATTA
B_2[127]_1[119]	GTCTGGCCTTCCTGTTCATATGT
B_32[135]_34[136]	CCTCATAACCCTCAGAACCGCCTAATAAGT
B_40[167]_42[168]	CTTAAATCGCAAATCAGATATAGAAGGCATTT
B_9[120]_7[119]	AATTGCGGCGGGCCGTTTTCACTGCTCGTC
B_28[63]_27[63]	AAATTGGGCTTGAGATGACGTTGGGAAGAAAA
B_36[63]_35[63]	CCTTATTAGCGTTTGCAGAACCGCCACCCTCA
B_16[95]_29[159]	TATCTTTATTTGGAACGAGCAGACGGTCAATCATA
B_16[159]_37[159]	CAATCAATTTTCAGAAGGAAAGGTGAATTATCACC
B_2[63]_1[55]	ATTCTCCGTGGGAACATGCCTGAG
B_32[167]_34[168]	AACTACAACCCTCAGAGCCACCACACATGGCT
B_38[63]_3[159]	AAACGTAGTTTTTTCCCAGGGCTGCGCAACTGTTG
B_23[56]_22[40]	TAGTAGTAAAATTAATGCCGGAGAGGGTAGCT
B_4[71]_6[72]	CACCGGAAAAACTTAAATTTCTGGCAAGAA
B_38[39]_40[40]	GGTGGCAGCAGCCTTTACAGAGTATTTATC
B_28[159]_27[159]	CAAGAACCGGATATTCACATTCAACTAATGCA
B_30[71]_32[72]	AATACGTAAAAGGAGCCTTTAATTGTATGGGA
B_1[152]_2[168]	ACAGGAAGATTGTATAAGCAAATATCAGCT
B_41[152]_39[159]	AATAGCAAAAGATTAGTTGCTATTGATAACCCACAAGAAT
B_22[167]_24[168]	AATGCAATAAACATTATGACCCTGAATATAAT
B_4[167]_6[168]	TGGGTAACTAGTGATGAAGGGTAGTCGCTG
B_30[39]_32[40]	AAAACGACACGTTGAAAATCTCTTCAGCGG
B_24[159]_23[151]	TCAACATGTTTTAAATAAATCGGT
B_21[88]_19[87]	TATCATATTGCAAATCCAATCGCATTTTAATG
B_20[71]_21[87]	GAACGCGAATTACTAGAAAAAGCCTGTTTAG
B_1[19]_2[40]	TTTTGATCTACAAAGGCTATCAGGTCATAACGGCGG
B_32[63]_31[63]	AACAACTTTCAACAGTCAAAAAAAAGGCTCCA
B_4[135]_6[136]	TTGTAAAAAATCCCGTAAAAAAAGTCATAACG
B_14[39]_16[40]	CATTCTGGACAATATTTTTGAATGAATACATT
B_8[103]_10[104]	CGGGGGTTCTAATGAGTGAGCTAATGAGAGAG
B_4[39]_6[40]	ATGTTTACTCACGGAAAAAGAGACTGCCATCC
B_36[95]_35[87]	GCGTTTTCATCGGCAAGCCGCC
B_23[120]_22[104]	ATAAAGCCGGGTGAGAAAGGCCGGAGACAGTC
B_38[95]_7[159]	ATTAAGACTTTCTGTGGTGGTTCAGCAAATCGTTA

B_11[120]_9[119]	CGTGAACTTCACCGCCTGGCCCCTCACATT
B_34[167]_36[168]	TTTGATGAATTCACAAACAAATAAGTCACCAA
B_36[39]_38[40]	CATAATCTTGTCACAATCAATATACATAAA
B_16[127]_33[159]	GTTGAAATTTTGTACTGGACCCTCAGAACCGCCA
B_12[135]_14[136]	GGCGCTAGCATCACGCAAATTAACGAAAAACG
B_12[103]_14[104]	CGCTGCGCTCAGTGAGGCCACCGAACGCTCAA
B_16[167]_18[168]	CAAACCCTCTGATTGTTTGGATTTAAAACA
B_24[39]_26[40]	AATGGTCTGACTATTATAGTCAGCTTTAAA
B_2[71]_4[72]	CCCGTCGGCAGGAAGATCGCACTATAACCT
B_14[103]_16[104]	TCGTCTGATAAAAATACCGAACGAGGAAGGTT
B_34[135]_36[136]	TTTAACGGGTTGAGGCAGGTCAAATCAGTA
B_14[135]_16[136]	CTCATGGATAAAACAGAGGTGAGGAGTTGGCA
B_7[88]_5[95]	GCTGGTAACTGGTCAGCAGCAACCCTCATTTGCCGCCAGC
B_30[167]_32[168]	AGCGAAAGACCATCGCCCACGCATCCAGTACA
B_13[88]_11[87]	TTTTATAAGTAACCACCACACCCGCAAAGGGC
B_38[71]_40[72]	ATGTTAGCAGGGAAGCGCATTAGATAATTTGC
B_32[159]_31[159]	CGCCTGTAGCATTCCAGCCGACAATGACAACA
B_30[159]_15[95]	ACAGCATCTTTGGAGCACTCCTAAAACATCGCCAT
B_21[120]_19[119]	TAAAGCCGTTGGGTTATATAACGTGAGTGA
B_34[39]_36[40]	TTAAGAGAGAGCCGCCACCCTCCATCTTTT
B_10[39]_12[40]	GAAATCGGCCAGTTTGGAACAAGACTATGGTT
B_16[63]_25[159]	AGAGCCGTTTTGCAAAAGAAAGCAAACTCCAACAG
B_40[71]_42[72]	CAGTTACACAAGAACGGGTATTAAAATGCAGA
B_26[39]_28[40]	CAGTTCACATTATACCAGTCAGGGTTTAAT
B_8[167]_10[168]	TGCGCGCCCCTGTCGTGCCAGCTGGGTGGT
B_12[159]_37[127]	GAAGGGAATTTGGAATACCGAAGGTAAATATTGAC
B_4[103]_6[104]	GAGGTGGAAGTTGGGCGGTTGTGTTCAGCGTG
B_22[135]_24[136]	TTCAAAATCAGAGCATAAAGCTATGCAACT
B_29[88]_27[95]	ATAAATTGTGCCCTGACGAGAAACGAACTAACGGAACA
B_36[127]_35[119]	AATCAAGTTTGCCTTTGCCAGCAT
B_20[39]_21[55]	GTTAATTTGGCGTTAAATAAGAATAAACACCG
B_18[167]_20[168]	GAAATAAATCCCTTAGAATCCTTATCAAAA
B_28[167]_30[168]	AATCTTGAAGGGAACCGAACTGACCCCTCAGC
B_14[95]_13[87]	AATGGATTATTTACATAGAAGTGT
B_24[127]_23[119]	GGTGTCTGGAAGTTTCATTAAGCA
B_42[71]_41[55]	ACGCGCCTGTTTATCAACAATAGATCTTTCCT
B_4[159]_37[63]	GCCAGGGTTTTAAAATACAGAAAATTCATATGGTT
B_30[63]_3[95]	ATGCCACTTTTGGGAACGGCCAGCCAGCTTTCCGG
B_24[63]_23[55]	TTTGACCATTAGATACTCTACTAA
B_26[95]_7[63]	CCAATACTTTTTCCTCACAGCGCGGTTGCGGTATG

B_35[88]_33[87]	ACCAGAACAACAGTTAATGCCCCTAGCCCG
B_32[39]_34[40]	AGTGAGAGCTCAGTACCAGGCGTGAAAGTA
B_41[88]_39[95]	ACCGCACTCGTCTTTCCAGAGCCCGGGAGAATTAACTG
B_9[88]_7[87]	TGGGGTGCTCTGCCAGCACGCGTGGCCCTGCG
B_14[71]_16[72]	TTCACCAGGCGCGAACTGATAGCAACAACT
B_4[127]_33[63]	CGACGGCTTTCTGAAACAGATAAGTGCCGTCGAG
B_31[120]_29[119]	AACAGCTTAGAGGCTTTGAGGACTCTGCTCCA
B_22[71]_24[72]	TAGCTGATGCATTAACATCCAATAGAACGAGT
B_12[167]_14[168]	CGAGAAAGCTTTGATTAGTAATAAGAACAA
B_4[95]_29[63]	GCCGCCACTTTACGAAGGCCGCGAAACAAAGTACA
B_14[63]_13[63]	TCACACGACCAGTAATTTTTAGACAGGAACGG
B_19[120]_17[127]	ATAACCTATGAATATACAGTAATCATCATATTCCTGA
B_2[135]_4[136]	TAATTCGCCGCCATTCGCCATTCATCACGACG
B_36[71]_38[72]	CATAGCCCTACCAGCGCCAAAGACTACGCAGT
B_33[120]_31[119]	GTACCGCCGTTAGCGTAACGATCTATTTCTTA
B_36[135]_38[136]	GCGACAGGGAAATTATTCATTAAACCGAGG
B_26[71]_28[72]	ATATTCATATCTACGTTAATAAAACACCAGAA
B_26[167]_28[168]	ATAGCGAGGATACATAACGCCAAATCAAGAGT
B_6[39]_8[40]	CACGCAACGCACTCAATCCGCCGGGTTGAGGA
B_35[120]_33[119]	TGACAGGAGGGTCAGTGCCTTGAGGAGGTTTA
B_38[127]_11[159]	ATAATAACTTTGAAAGCGATTTTTTGGGGTCGAGG
B_16[71]_18[72]	AATAGATTCATTATCATTTTGCGGATTCGC
B_42[135]_41[119]	ACAAAAGGTAAAGTAATTCTGTCGCCGTTTT
B_42[167]_41[151]	TCGAGCCAGTAATAAGAGAATATACCGCGCCC
B_8[159]_37[95]	TGTGCACTTTTTCCTTATAAAAGGGCGACATTC
B_36[167]_38[168]	TGAAACCAGTCACCGACTTGAGCCAAGCAGAT
B_18[71]_20[72]	CTGATTGCCATTTGAATTACCTTAGACAAA
B_24[167]_26[168]	GCTGTAGCGTCAGGATTAGAGAGTAAACCAAA
B_36[159]_35[159]	TCGATAGCAGCACCGTGACGATTGGCCTTGAT
B_22[39]_24[40]	ATTTTTGAAAGGTGGCATCAATATTTCGCA
B_10[167]_12[168]	TTTTCTTTTGCCGTAAAGCACTAAACGTGG
B_18[135]_20[136]	AACGTCAGTGCTTCTGTAAATCGTTTAACCTC
B_20[135]_21[151]	CGGCTTAGAACGCTCAACAGTAGGGCTTAATT
B_24[95]_23[87]	GATTCCCAATTCTGCAATCATA
B_34[159]_15[127]	TACAGGAGTTTGGAATTGAACCACCAGCAGAAGA
B_4[63]_25[63]	ACAATCGGTTTCCTCAAATGAAGCAAAGCGGATTG
B_10[63]_9[63]	AAAATCCTGTTTGATGCCACACAACATACGAG
B_8[63]_25[95]	CGTGAGCCTTTGCGGAATAGGAAGCCCGAAAGA
B_10[159]_9[159]	TCACCAGTGAGACGGGCTTTCCAGTCGGGAAA
B_8[135]_10[136]	AGAATGCGTTGCGCTCACTGCCCGCAACAGCT

B_30[135]_32[136]	CGGCTACGATACCGATAGTTGCCAGACAGC
B_2[159]_1[151]	AACCAATAGGAACGCCCCCCAAAA
B_12[63]_25[127]	CGCTACAGTTTTAAAATGTTTAATTCGAGCTTCAA
B_40[135]_42[136]	AGCTACATCGTAGGAATCATTAAAGTACCG
B_12[39]_14[40]	GCTTTGACAGGCCGATTAAAGGGAAAAAGGGA
B_7[120]_5[127]	ATAAACACGGACTTGTAGAACGACATCGACATAAAAA
B_14[127]_13[119]	AATACCTACATTTTGGTAAAAGA
B_14[159]_13[159]	CCAGCCATTGCAACAGCGTTGTAGCAATACTT
B_26[127]_11[63]	GGTAATAGTTTGGCGCGTAGTCCACTATTAAAGAA
B_31[88]_29[87]	TTTATCAGGGAAGTTTCCATTAATCGCCTG
B_10[135]_12[136]	GATTGCCCCATCACCCAAATCAAGAAGGAGCG
B_6[135]_8[136]	GAACGTGCTCCCTTACACTGGTGTCTGCGGCC
B_28[39]_30[40]	TTCAACTAGCGATTATACCAAGACCAACCT
B_14[167]_16[168]	TATTACCGCGCCTGCAACAGTGCCAAATAT
B_26[63]_3[63]	TGAATCCCTTTCGAAACGTACGACAGTATCGGCCT
B_26[159]_15[63]	AGGCTTTTTTTCAATAGATGCTATTAGTCTTTAAT
B_23[152]_22[136]	TGTACCAAGCCTGAGTAATGTGTAGGTAAAGA
B_28[71]_30[72]	CGAGTAGTACGGAGATTTGTATCAACGGGTAA
B_16[135]_18[136]	AATCAACATTATCAGATGATGGCATCAGGTTT
B_12[71]_14[72]	TAATGCGCTACGCCAGAATCCTGTGGCAGA
B_33[88]_31[87]	GAATAGGTGTAAATGAATTTTCTGTATCGG
B_24[135]_26[136]	AAAGTACAGCGAACCAGACCGGAGTTTTGC
B_18[39]_20[40]	CGCAGAGGAAGAAAACAAAATTAAATATTTTA
B_2[167]_4[168]	CATTTTTTGGAAGGGCGATCGGTATTAAGT
B_38[159]_15[159]	AAAGTTACTTTATCTGGTCCGGTCAGTATTAACAC
B_23[88]_22[72]	CAGGCAAGATCAATATGATATTCAACCGTTC
B_42[103]_41[87]	GACAATAAACAACATGTTCAGCTACCAAGT
B_20[167]_21[187]	TCATAGGTCCATATTTAACAACGCCAACATGTTTT
B_26[135]_28[136]	CAGAGGGAGATTTAGGAATACCATTACCCA
B_20[103]_21[119]	AATGCTGAGCGTTATACAAATTCTTACCAGTA
B_29[120]_27[127]	TGTTACTTGTAACAAAGCTGCTCAAAAGATTCATCAGTTG
B_6[103]_8[104]	GTGCTGGTTGGGTAAAGGTTTCTTGGTCATAC
B_40[103]_42[104]	CTAACGAGCATCGAGAACAAGCAACAGACGAC
B_38[167]_40[168]	AGCCGAACTGAGTTAAGCCCAATATTTGAAGC
B_16[103]_18[104]	ATCTAAAAAGAAGGAGCGGAATTACAGTACCT
B_18[103]_20[104]	TTTACATCACATAAATCAATATATTATATGTA
B_24[71]_26[72]	AGATTTAGCATCAAAAAGATTAAGCGTCATAA
B_13[120]_11[119]	GTCTGTCGGCGCTGGCAAGTGTGCCCACTA
B_6[167]_8[168]	GCAGCCTCACGGCATCAGATGCCGTGTCAC
B_1[56]_2[72]	AGTCTGGAGCAAACAAGAGAATCGTAACAA

B_28[135]_30[136]	AATCAACAGCCGGAACGAGGCGGGTAGCAA
B_10[71]_12[72]	AGCAGGCGCGTGGACTCCAACGTCCGCGCT
B_38[135]_40[136]	AAACGCACGCTAATATCAGAGATTGCACCC
B_19[88]_17[95]	GAAACAGTGGGAGAAACAATAACGGAACAAAGAAACCACC
B_1[88]_2[104]	GTAATCGTAAAACTAGCATGTCAAAGCCAGCT
B_16[39]_18[40]	TGAGGATTCCGAACGTTATTAATTCAAAATCG
B_2[95]_1[87]	CATTAAATGTGAGCGAGATGAACG
B_21[56]_19[55]	GAATCATAGAAAACTTTTTCAAATTTACATTT
B_34[71]_36[72]	TTCGGAACGAGCCACCACCCTCAGTTTTCGGT
B_32[71]_34[72]	TTTTGCTAAGGGTTGATATAAGTACTGCCTAT
B_34[63]_3[127]	CTATTATTTTTCAGTGCCAGGAAACCAGGCAAAG
B_8[71]_10[72]	TTCGCGTCCCGGAAGCATAAAGTTTGCCCC
B_41[120]_39[127]	TATTTTCAATTTTATCCTGAATCTTCAGAGGGTAATTGAG
B_10[103]_12[104]	TTGCAGCAGTCTATCAGGGCGATGAGCGGTCA
B_1[120]_2[136]	ACCCCGGTTGATAATCAGAAAAGATCAAAAA
B_6[71]_8[72]	TGCCAACGAGCCGGGTCACTGTTCCTGTTC
B_6[159]_5[159]	CGGCCAGAGCACATCCCCGCACAGGCGGCCTT
B_19[56]_17[63]	AACAATTTTTTGAATACCAAGTTATTAAAAGTTTGAGTAA
B_19[152]_17[159]	ATTAATTTGAAATTGCGTAGATTTATTCATCAATATAATC
B_6[63]_5[63]	GCAGCACCGTCGGTGGGCAGAAACAGCGGATC
B_30[103]_32[104]	TTTCATGACTTGCTTTCGAGGTGAAAAGTTTT
B_22[103]_24[104]	AAATCACCGCAAAGAATTAGCAAAATTCCATA
B_28[103]_30[104]	ATAAGGCTTGTCGAAATCCGCGACAAAGACTT
B_32[103]_34[104]	GTCGTCTTTCCAGACGTTAGTATCACCGTACTCAGTAACAGTG
B_36[103]_38[104]	ACTGTAGCAACCGATTGAGGGAGGCAAAAGAA
B_38[103]_40[104]	CTGGCATGAACACCCTGAACAAAGTACCAACG
B_26[103]_28[104]	GATAGCGTACATTATTACAGGTAGTTCAGTGA
B_24[103]_26[104]	TAACAGTTCTTCAAATATCGCGTTTTAGACTG
B_34[103]_36[104]	CCCGTATACACCACCAGAGCCGCCAGCGTCAG

Table S2: Modified strands used in the DNA origami. Staple strand positions are given as B_H5′[p5′]_H3′[p3′], where the first helix number and bracketed index specify the staple's 5′ start (helix number and position within that helix), and the second pair specifies the staple's 3′ end. Sequences are listed in the 5′ to 3′ direction. Regions corresponding to the main double-layer structure are shown in black; thymidine spacers in yellow; transient binding regions in red; hinge sequences in blue; and fluorophore modifications in dark red.

Position	Name	Sequence
B_2[103]_4[104]	Binding_site_1	AGTCCTATTTTCATCAACACCGCTTCTGGTGCCAGCTTTCA
B_8[39]_10[40]	Binding_site_2	AGTCCTATTTCCCCGGGTTATCCGCTCACAATTGTGGTTCC
B_2[39]_4[40]	Static_fluorophore	/Atto647N/TTTTATTGACCGCCAGTTTGAGGGGACGACAGCGCC
B_11[88]_9[87]	Hinge_base	GAAAAACCAGCGGTCCACGCTGGTGTAAAGCCTTTGACACTTCGTGTGTG
Hinge	Hinge_binding_site	CGTGGATCACACGAAGTATCACACACGAAGTGTTTAGGACT
Hinge	Mobile_fluorophore	/Atto647N/TACACTTCGTGTGTGATACTTCGTGTGATCCACGCACACACGAAGTGTC

Movie S1: Nonstop tracking of two fluorophores at the nanoscale. Animation of the co-tracking of two randomly moving fluorophores at the nanometer scale. The latest positions of the two fluorophores are indicated by the yellow stars. Heatmaps show the aggregated localizations and reveal the underlying structure of the DNA construct with its three states: two bound states and one unbound, freely-diffusing state.

SI References

1. Kopperger, E. et al. A self-assembled nanoscale robotic arm controlled by electric fields. Science 359, 296–301 (2018).
2. Dreher, Y. et al. Genotype-phenotype mapping with polyominos made from DNA origami tiles. Biophys. J. 121, 4840–4848 (2022).
3. Douglas, S. M. et al. Rapid prototyping of 3D DNA-origami shapes with caDNAno. Nucleic Acids Res. 37, 5001–5006 (2009).
4. Nečas, D. & Klapetek, P. Gwyddion: an open-source software for SPM data analysis. Open Phys. 10, 181–188 (2012).
5. C. Truong, L. Oudre, N. Vayatis. Selective review of offline change point detection methods. Signal Processing, (2020).
