# Supplementary figures and images for: Uninterrupted optical resolution of identical point scatterers undergoing nanometric changes in distance
Source: Proc Natl Acad Sci U S A. 2026 Jul 13;123(29):e2604558123. doi: 10.1073/pnas.2604558123 (PMC13389594; doi:10.1073/pnas.2604558123)

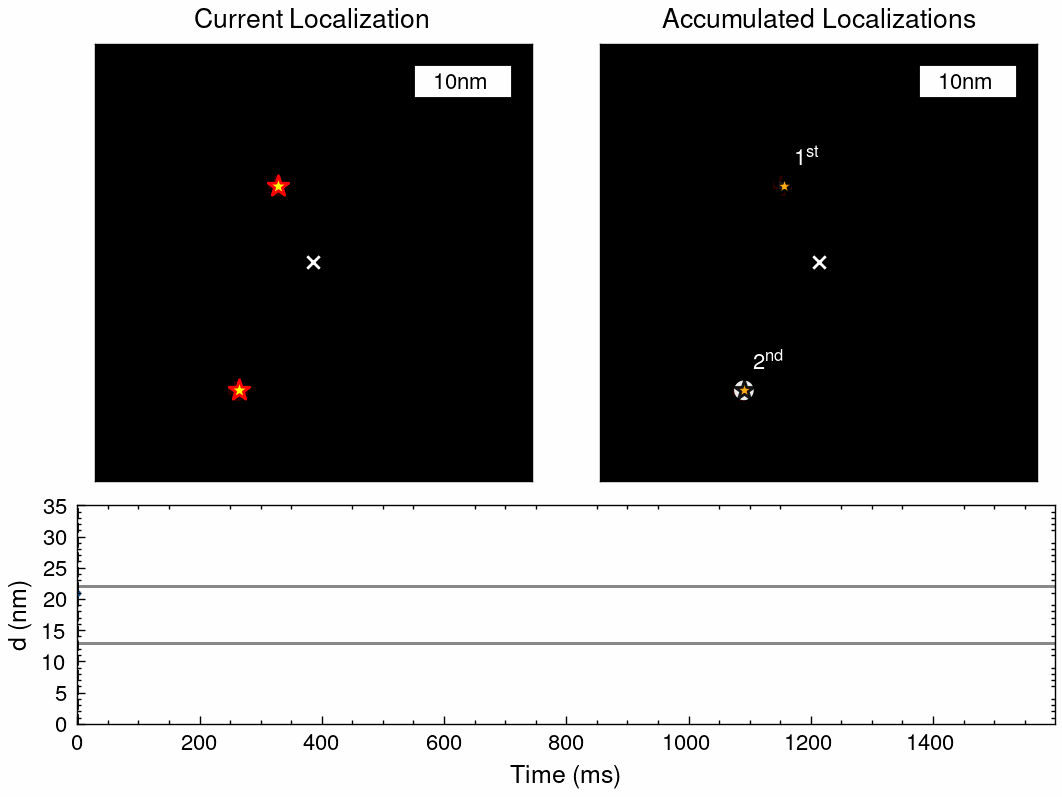

Supplement: Movie S1. — Nonstop tracking of two fluorophores at the nanoscale. Animation of the co-tracking of two randomly moving fluorophores at the nanometer scale. The latest positions of the two fluorophores are indicated by the yellow stars. Heatmaps show the aggregated localizations and reveal the underlying structure of the DNA construct with its three states: two bound states and one unbound, freely-diffusing state. [file pnas.2604558123.sm01.gif]
